# CAR T-Cell-Based gene therapy for cancers: new perspectives, challenges, and clinical developments

**DOI:** 10.3389/fimmu.2022.925985

**Published:** 2022-07-22

**Authors:** Manasi P. Jogalekar, Ramya Lakshmi Rajendran, Fatima Khan, Crismita Dmello, Prakash Gangadaran, Byeong-Cheol Ahn

**Affiliations:** ^1^ Helen Diller Family Comprehensive Cancer Center, University of California San Francisco, San Francisco, CA, United States; ^2^ Department of Nuclear Medicine, School of Medicine, Kyungpook National University, Kyungpook National University Hospital, Daegu, South Korea; ^3^ Department of Neurological Surgery, Feinberg School of Medicine, Northwestern University, Chicago, IL, United States; ^4^ BK21 FOUR KNU Convergence Educational Program of Biomedical Sciences for Creative Future Talents, Department of Biomedical Science, School of Medicine, Kyungpook National University, Daegu, South Korea

**Keywords:** immunotherapy, gene therapy, CAR T-cell therapy, solid cancers, hematologic malignancies

## Abstract

Chimeric antigen receptor (CAR)-T cell therapy is a progressive new pillar in immune cell therapy for cancer. It has yielded remarkable clinical responses in patients with B-cell leukemia or lymphoma. Unfortunately, many challenges remain to be addressed to overcome its ineffectiveness in the treatment of other hematological and solidtumor malignancies. The major hurdles of CAR T-cell therapy are the associated severe life-threatening toxicities such as cytokine release syndrome and limited anti-tumor efficacy. In this review, we briefly discuss cancer immunotherapy and the genetic engineering of T cells and, In detail, the current innovations in CAR T-cell strategies to improve efficacy in treating solid tumors and hematologic malignancies. Furthermore, we also discuss the current challenges in CAR T-cell therapy and new CAR T-cell-derived nanovesicle therapy. Finally, strategies to overcome the current clinical challenges associated with CAR T-cell therapy are included as well.

## 1 Cancer immunotherapy

The immune component plays a critical role in maintaining a balance between recognizing cancer cells as foreign bodies and showing tolerance towards self-antigens. The cancer immunity cycle depends on the ability of T-cells to attack and eliminate cancer cells. Antibodies against PD-1 and PD-L1 have significantly improved the outcomes of patients with melanoma and lung cancer (1, 2).

Cancer immunotherapy relies on the immune system of patients to recognize and attack cancer cells. Cancer immunotherapies potentiate immune cells by relieving their suppression or directly activating them to perform their immune function more effectively. There are different cancer immunotherapies based on the targeted immune components.

### 1.1 Cytokines

In the 1970s, tumor necrosis factor (TNF) was systemically injected into patients with cancer as a cancer immunotherapy modality. However, toxicities due to TNF infusion, such as fever, rigors, and pulmonary edema, limited its use in cancer treatment (3). Interleukin 2 (IL-2) is another cytokine that demonstrated efficacy and was approved by the Food and Drug Administration (FDA) for metastatic renal cell cancer in 1992 and metastatic melanoma in 1998. However, similar to TNF, the use of IL-2 was limited due to the severe toxicities it induced in the patients, which outweighed the benefits of the treatment (4).

### 1.2 Vaccines

The Bacillus Calmette-Guerin (BCG) vaccine was the first vaccine approved by the FDA in 1990 for the treatment of superficial bladder cancer. In 2010, the FDA approved a sipuleucel-T vaccine for castrate-resistant prostate cancer to extend the overall survival of patients. However, these vaccines failed to confer durable responses (5). This was perhaps due to the limited knowledge on dosing, vaccine availability in the tumor microenvironment, and engagement of T cells.

### 1.3 Checkpoint inhibitors

The discovery of immune checkpoint inhibitors was a breakthrough in cancer research. Allison showed that blocking cytotoxic T lymphocyte antigen 4 (CTLA-4) releases the brake on the immune system and boosts the immune response against cancer cells (6). Ipilimumab, a CTLA-4 checkpoint inhibitor, significantly improves survival in patients with metastatic melanoma (7). The CTLA-4 receptor is induced on T cells 48-72 h after T-cell receptors are engaged with antigen-presenting cells. The CTLA-4 receptor is also expressed on FOXP3 positive regulatory T cells (8). Mechanistically, CTLA-4 is known to have a PI3K-like motif, implying that it may interact with the PI3K, MAPK, and NF-kB pathways (9). Following CTLA-4 treatment, the FDA approved the inhibition of programmed death-1 (PD-1) and its ligand PD-L1 as immune checkpoint inhibitors for metastatic melanoma and lung cancers (10). PD-1 and PD-L1 interactions regulate immune escape in the tumor and tumor microenvironment. PD-1 expression on T-cells is a marker of antigen-experienced exhausted T-cells (11). Mechanistically, ligation of TCR and PD-1 leads to phosphorylation of a tyrosine residue located within the immunoreceptor tyrosin-based switch motifs (ITSM) of the PD-1 cytoplasmic tail. These events, including binding of phosphatases and augmentation of PTEN, expression contribute to decreased T-cell proliferation, survival, protein synthesis, and IL-2 production (12). An increasing number of clinical trials are being launched every year using these checkpoint inhibitors as monotherapies or in combination with standard of care or targeted therapies for various malignancies.

### 1.4 Adoptive cell therapy

CAR T-cell therapy is an adoptive cell-transfer-based immunotherapy developed by genetically modifying T cells. CAR T-cell therapy is directed against tumor-associated antigen and is independent of MHC-receptor presentation by the. This therapy has revolutionized the treatment of patients with B-cell lymphomas by conferring durable clinical responses. Several ongoing clinical trials have tested the efficacy of CAR T-cell therapy for different malignancies (13).

## 2 Genetic engineering of T-Cells

**Figure 1:**
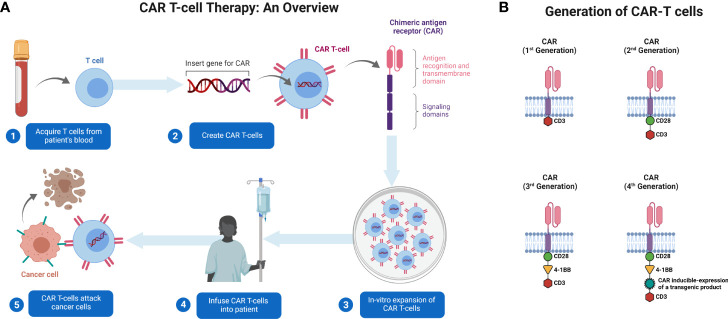
Generation and administration of CAR T-cells in patients with cancer. **(A)** T cells are collected from patients’ blood *via* apheresis. They are genetically engineered to express CAR and cultured *ex vivo* for expansion. CAR T-cells are then administered to patients. The cells identify their target and kill the tumor cells expressing that target. **(B)** Illustration of basic structure of four generations of CAR T-cells. Created with BioRender.com.

The source of T cells for CAR T-cell production can be either the patient (autologous) or a donor (allogenic). Blood is collected by venipuncture or apheresis from the patient and donor. The T cells undergo purification and are subjected to genetic engineering (14). CARs are artificially generated receptors that have been built to specifically target antigens expressed on the cell surface (15). T cells are typically engineered to express CARs by transducing patient T cells with a virus that encodes aDNA construct. The resulting CAR T cells are then expanded ex vivo and infused back into the patient (**Figure 1A**). Genetic engineering is performed using viral or non-viral methods to eliminate the expression of proteins such as HLA class I and II, in allogeneic T cells (16). This helps mitigate rejection by the hosts’ immune system. These vectors are also co-delivered with transposase to enable the integration of transgenes into the genome in a random fashion (17). Transgenes are typically introduced under the control of endogenous promoters. A typical CAR consists of a single-chain variable fragment (scFv) with a flexible hinge domain, transmembrane domain, and CD3ζ activation domain (14) (**Figure 1A**) and several CAR T-cell generations have been engineered (18) (**Figure 1B**). The key raw material for CAR T-cell products is the viral vector. The viral vector is stored in large quantities at −80°C for up to 9 years (19). Safety, sterility, titer, purity, and potency of the vector are crucial for infusion into patients (20). Lentiviral and retroviral vectors are potentially oncogenic however, vectors are associated with a lower risk of mutagenesis (21). It is also important to increase the safety of CAR T-cell therapy to improve the specificity of modified T cells.

## 3 CAR T-Cell therapy

### 3.1 Solid tumors

**Figure 2:**
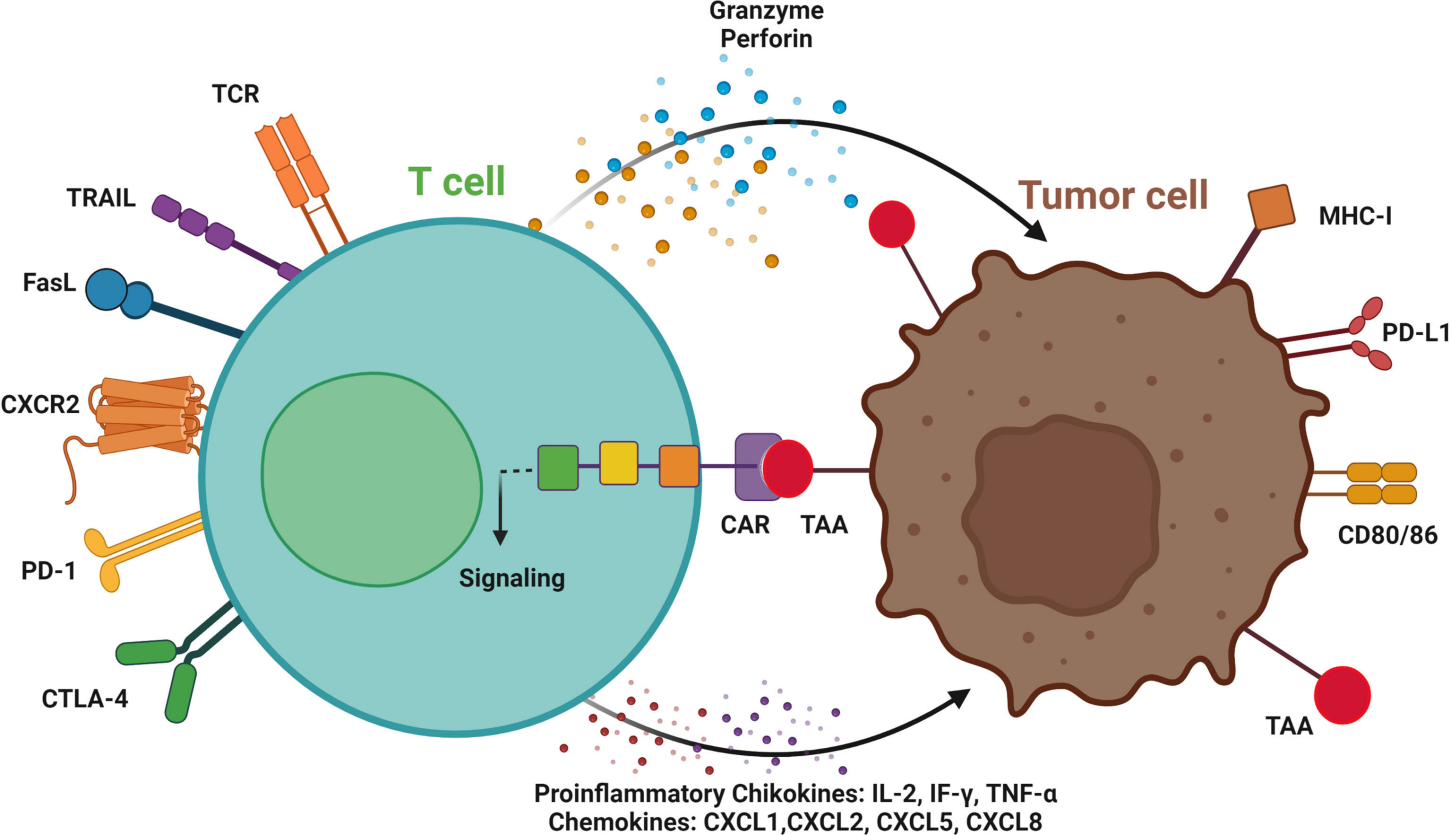
T cell-mediated antitumor effects by chimeric antigen receptors (CAR). Engineered CAR T-cells can recognize tumor cells by CAR binding to tumor-associated antigen (TAA), signaling activation and targeting the tumor cells by secreting granzymes, and perforins, and inducing TRAIL and FasL expression. CAR T-cells can be used as an ideal platform to deliver immune checkpoint therapeutic antibodies, such as anti-PD1 and CTLA-4 antibodies. CC-chemokine receptor 2; CD, cluster of differentiation; CTLA-4, cytotoxic T-lymphocyte associated protein 4; MHC, major histocompatibility complex; PD-1, programmed cell death protein-1; PD-L1, programmed death-ligand 1; and TCR, T cell receptor. Immune cells invade the tumor by activating proinflammatory cytokines and chemokines. Created with BioRender.com.

Tumors can suppress T-cells activity through various methods, and several studies have examined engineering cells to overcome this suppression. We evaluated clinical trials for the adequacy of CAR T-cell therapies in solid tumors (**Table 1**) and important targeted surface markers (**Figure 2**).

**Table 1:** Ongoing and currently recruiting clinical trials involving CAR T-cell therapies for solid tumors (22).

Intervention	Condition	Location	ClinicalTrials.gov Identifier
CEA CAR T-cells	Pancreatic Cancer	Chongqing University Cancer Hospital Chongqing, Chongqing, China	NCT04348643
CD276 CAR T-cells	Advanced Pancreatic Cancer	Li Yu Shenzhen, Guangdong, China	NCT05143151
CT041 autologous CAR T-cell	Pancreatic Cancer	Anhui Provincial Cancer Hospital Hefei, Anhui, China	NCT04581473
BPX-601 CAR T-cells	Metastatic Castration-resistant Prostate Cancer, Metastatic Prostate Cancer, Metastatic Pancreatic Ductal Adenocarcinoma, Metastatic Pancreatic Cancer and Metastatic Pancreatic Adenocarcinoma	Moffitt Cancer Center Tampa, FL, USA	NCT02744287
Anti-hCD70 CAR transduced PBL	Pancreatic Cancer	National Institutes of Health Clinical Center Bethesda, MD, USA	NCT02830724
CEA CAR T-cells	Breast Cancer	Chongqing University Cancer Hospital Chongqing, Chongqing, China	NCT04348643
4S CAR T-cells	Breast Cancer	The Seventh Affiliated Hospital, Sun Yat-Sen University Shenzhen, Guangdong, China	NCT04430595
CD44v6-specific CAR T-cells	Cancers Which Are CD44v6 Positive	Shenzhen Children’s Hospital, Shenzhen, Guangdong, China	NCT04427449
Anti-hCD70 CAR transduced PBL	Breast Cancer	National Institutes of Health Clinical Center, Bethesda, MD, USA	NCT02830724
AIC100 CAR T-cells	Anaplastic Thyroid Cancer and Relapsed/Refractory Poorly Differentiated Thyroid Cancer	Weill Cornell Medical College New York, NY, USA	NCT04420754
single dose of CAR T-GFRa4 cells	Metastatic Medullary Thyroid Cancer	University of Pennsylvania Philadelphia, PA, USA	NCT04877613
EGFRv III -CAR transduced PBL	Malignant Glioma	National Institutes of Health Clinical Center, 9000 Rockville Pike Bethesda, MD, USA	NCT01454596
anti-CD133-CAR vector-transduced T cells	Brain Tumor	Biotherapeutic, Department and Pediatrics Department of Chinese PLA General Hospital Beijing, Beijing, China	NCT02541370

CAR T, chimeric antigen receptor-T; CAE, carcinoembryonic antigen; CD276, cluster of differentiation 276; CT 041, claudin 18.2; BPX-601, PSCA-Targeted CAR T-Cells; hCD70, human cluster of differentiation 70; 4S CAR T, fourth-generation safety-designed CAR; GFRA4, GDNF Family Receptor Alpha 4; EGFR, epidermal growth factor receptor and CD133, cluster of differentiation 133.

#### 3.1.1 Pancreatic tumor

CAR T-cells have demonstrated therapeutic efficacy both *in vitro* and in orthotopic or metastatic xenograft mouse models. Studies have hypothesized that chemokine receptors CXCR2-expressing CAR T-cells could traffic towards IL-8 more efficiently. In xenograft animal models, CAR T-cells expressing CXCR2 showed significant antitumor activity against αvβ6-expressing pancreatic tumors (23). Interestingly, 4-1BB co-stimulation can lower PD-1 expression in the generated T cells, showing more potent antitumor activity against PD-L1-expressing tumor cells (24, 25). Additionally, clinical trials for pancreatic, colorectal, and hepatocellular carcinomas demonstrated the inhibitory effect of CD133-CAR T-cells on the metastatic potential of the cancers (26). In addition, other varieties of antigen targets for pancreatic cancer CART-cell therapy, such as CD24 (27), MUC-1 (28), PSCA (29), mesothelin (30), and FAP (31), have been investigated in preclinical studies and clinical trials.

#### 3.1.2 Breast cancer

Several studies have shown that, CAR T-cells are very potent at killing triple-negative breast cancer (TNBC) tumor cells in an exceedingly tMUC1-highly specific manner. MUC28z CAR T-cells, a specifically contain CAR with both CD3ζ and CD28 signaling domains, which increases the synthesis of cytokine IFN-γ, granzyme B, and other kinds of cytokines or chemokines produced by Th1 cells. In addition, a single dose of MUC28z CAR T-cells could significantly abolish TNBC cell proliferation and increase survival benefits in xenograft models (32). Another study revealed that 4-1BB or CD27 co-stimulation enhanced NKG2D CAR T-cells involved in anticancer function in TNBC tumor models (33). Another study showed that CAR T-cells support HRG1β to successfully abolish breast cancer cell proliferation through HER family receptors and deliver a practical therapeutic approach to overcome cancer resistance, specifically against HER2-based targeted therapy (34). Human anti-HER2 CAR T-cells also exhibit desirable targeting, triggering cell death in HER2 overexpressing breast cancer cells (35). Furthermore, another biomarker, mesothelin, identified by special CAR T-cells, has been reportedly as promising in immunotherapy for breast cancers (36).

#### 3.1.3 Thyroid cancer

The first study on CAR T-cell therapy for advanced thyroid cancer revealed the development of an intercellular adhesion molecule 1 (ICAM 1)- specific CAR T-cell and its preclinical efficacy (37). However, various factors may impede clinical translation of anti-ICAM 1-CAR T-cells. While T cells upregulate ICAM 1 expression and are followed by activation (38), it is possible that anti-ICAM 1-CAR T-cells might target each other, resulting in poor *in vitro* proliferation and persistence in patients with thyroidcancer. Another condition reported was elevated soluble ICAM 1 found in the serum of patients with thyroid cancer (39), which might neutralize anti-ICAM 1-CAR T-cells in the periphery before recognizing ICAM 1^+^ tumor cells. In the absence of a tumor-associated antigen target (TAA), alternative technologies using antibody-based CARs to mimic T cell receptor (TCR) recognition of specific tumor-neoantigens, such as the complex of BRAF^V600E^ oncoprotein with MHC, could be further investigated (40). The transgenic TCR tumor-infiltrating lymphocyte approach requires tumor cells to maintain the ability to process and present antigens at the cell surface. Medullary thyroid cancer (MTC) may be an excellent target for CAR T-cells therapies, given that these tumors commonly express carcinoembryonic antigen (CEA) and GDNF family receptor α4 (GFRA4). Indeed, GFRA4-specific CAR T-cell strategies are currently under preclinical development (41).

#### 3.1.4 Brain cancer

Various clinical studies have been completed and are ongoing using CAR T-cells in glioblastoma (GBM). The first clinical trial on humans involving 10 patients with recurrent GBM evaluated the effect of intravenously injected EGFRvIII-CAR T-cells; while CAR T-cells expanded within the blood and were trafficked to the tumor region, they found antigen loss in five out of seven patients, and therefore, the tumor microenvironment indicated higher expression of inhibitory molecules, and the rate of occurrence of Treg cells was higher, as indicated (42). Improve the CAR T-cell therapy requires identifying TAA expressed with stability and specificity with definite heterogeneity throughout the tumor region. An appropriate target was identified for these criteria. A study demonstrated *in vivo* therapeutic effects of intracranial delivery of chondroitin sulfate proteoglycan 4 (CSPG4)-CAR T-cells in nude mice transplanted with CSPG4-expressing glioma cells or GBM neurospheres models (43). As the endmost CAR T-cell product mixes with CD4^+^ and CD8^+^ CAR T-cells, this approach was refined to distinguish the T cell subsets that arbitrate antitumor activity. Another study revealed that the CD4^+^ CAR T-cell subset, was more effective than CD8^+^ CAR T-cells in orthotopic GBM mouse models and IL-13Rα2-CAR T-cells, which possibly indicated that CD8^+^ CAR T-cells were rapidly exhausted (44). Co-expression of the IL-8 receptor, CXCR1, and CXCR2, enhanced CAR T-cell trafficking and was stably retained at in the glioma tumor site in a mouse model (45). Genetically engineered EGFRvIII-CAR T-cells co-expressing a bispecific T-cell engager (BiTE) directed against EGFR (wild-type) were established in GBM tumor models (46). Additionally, various CAR target antigens in GBM tumors, including B7-H3 (47, 48), HER2 (49–51), and EphA2 (52), have been demonstrated in advanced phase I clinical trials using HER2-CAR T cells and in other preclinical studies (50, 53).

The development of a universal CAR T (UCAR T) cell, which allows a tri-cistronic transgene to encode three CAR molecules against HER2, IL-13Rα2, and EphA2, overcame the interpatient variability and targeted 100% of GBM tumor cells (54). In a different way to overcome antigen escape problems and tumor heterogeneity, a new CAR approach was designed that employs a toxin as the targeting entity, which was developed and tested in a murine glioma model. Chlorotoxin (CLTX) directed CAR T-cellsshowed GBM cell binding affinity by matrix metalloproteinase-2 and CLTX- CAR T-cells efficiently limited tumor growth in mouse model, which addressed the off-target effects (55) The ongoing and currently recruiting phase II clinical trials (thyroid tumor: I clinical trials) involving CAR T-cell therapies for solid tumors are listed in **Table 1**.

### 3.2 Hematologic malignancies

Hematologic malignancies, also known as blood cancers, arise from the uninhibited proliferation of abnormal blood cells and made up approximately 10% of all cancers in 2019 in the United States (56). CAR T-cell therapies have shown significant promise in the treatment of hematologic malignancies in recent years (57–61), although the first insight into their efficacy of CAR T-cell therapy was obtained from the clinical trials involving solid tumors (62, 63). The response time for CAR T-cell therapy is lower than that for other therapeutic strategies, such as tumor vaccines and immune checkpoint blockade, although this is not always true since, some of the CAR T-cells persist with a memory phenotype and respond more quickly (64, 65). These efforts have resulted in three FDA-approved first-of-their-kind therapies for treating refractory diffuse large B-cell lymphoma (DLBCL) and acute lymphoblastic leukemia (ALL) (66).

#### 3.2.1 Hodgkin’s lymphoma

Hodgkin’s lymphoma (HL) is less common than other hematologic malignancies, accounting for <1% of all cancers in the United States (56). In 2020, 83,087 new HL cases and 23,376 HL-related deaths were estimated worldwide (67). HL is characterized by Hodgkin Reed-Sternberg (HRS) cells belonging to the B-cell lineage. HRS and anaplastic large cell lymphoma (ALCL) cells highly express the cell surface marker CD30 (68). While the FDA-approved antibody-drug conjugate brentuximab vedotin is clinically effective in treating these tumors by targeting CD30 (68, 69), the progression-free survival (PFS) rate remains low at 5 years, suggesting that improved targeted therapies could cure the disease by driving tumor cells in long-term remission (70). CAR T-cell therapies directed towards CD30 have shown durable antitumor response in HL cell lines and mouse models (71, 72). Inducing expression of CCR4 in anti-CD30 CAR T-cells promotes their migration towards tumors in HL mouse xenografts (73). In phase I clinical trials, antitumor responses have been observed in the presence or absence of conditioning chemotherapy when patients with brentuximab-refractory HL and ALCL patients were treated with anti-CD30 CAR T-cells containing a CD28 (74) or 4-1BB costimulatory domain (75).

#### 3.2.2 Non-hodgkin lymphoma

Non-Hodgkin lymphoma (NHL) is more common than HL and constitutes approximately ~4% of all cancers in the United States (56). In 2020, 544,352 new NHL cases and 259,793 NHL-related deaths were estimated worldwide (67). NHL can be categorized as B-cell lymphoma (BCL) and T-cell lymphoma (TCL). Most BCL cells express the B-cell differentiation markers - CD19 and CD20, whereas some TCLs express the CD30 marker (76).

##### 3.2.2.1 B-Cell lymphoma

BCL constitutes the majority (~85%) of NHLs (77). DLBCL (26%), follicular lymphoma (FL; 13%), marginal zone lymphoma (MZL; 7%) and mantle cell lymphoma (MCL; 3%) are the main subtypes of NHL (76). CAR T-cell therapies targeting these antigens have shown a high overall response rate (ORR) and complete response rate (CRR) in NHL in clinical trials (60).

In a clinical trial involving seven patients, the City of Hope National Medical Center and Fred Hutchinson Cancer Research Center researchers used electroporation to introduce the CD20-specific CAR transgene into the T cells of patients with MCL and refractory BCL (78). This resulted in either stable disease (n=4) or partial response (n=1) or complete responses (n=2) with minimal toxicities (78). In another clinical trial published by the City of Hope, patients with recurrent DLBCL and refractory FL were treated with CD20- and CD19-specific CAR T-cells. Although minimal toxicity was observed, the persistence of infused cells remained low (79). The National Cancer Institute (NCI) first reported the efficacy of CD19-specific CAR T-cells incorporated with a CD28 costimulatory domain (FMC63-28Z) in combination with chemotherapy and IL-2 administration in the treatment of treating refractory FL and splenic MZL in a clinical setting (58, 80). While patients did not suffer from evident chronic toxicities, cytokine release syndrome (CRS) was observed (58). In a pilot study conducted by Till et al. (2012), patients with FL and MCL received CD20-specific CAR T-cells with costimulatory domains *via* electroporation followed by conditioning chemotherapy (81). Notably, patients showed partial or complete response and the persistence of T cells in the blood lasted for 9-12 months, which may be attributed to multiple IL-2 treatments (81). Another clinical trial involving the administration of anti-CD19 CAR T-cells in two children with relapsed and refractory (R/R) pre-B-cell ALL resulted in complete remission (82). Interestingly, one of the patients relapsed due to the emergence of CD19-negative cells, demonstrating a classic immune escape mechanism, indicating that and other B-cell markers are needed to improve the efficacy of treatment (82).

The NCI first reported successful administration of anti-CD19 CAR with a CD28 costimulatory domain in patients with DLBCL (83). Cyclophosphamide and fludarabine was included in their chemotherapy regimen prior to CAR T-cell infusion. The combination therapy worked well, driving refractory BCLs, including DLBCL, into complete remission (83). Another clinical trial demonstrated the efficacy of anti-CD19 CAR T cells containing CD28 and TCR zeta domains with reversible toxicities, when administered to children and young adults with relapsed or refractory B-cell ALL (B-ALL) following the aforementioned chemotherapy regimen (83, 84). Antitumor responses have also been observed when anti-CD19 CAR T-cells with a 4-1BB costimulatory domain were administered to patients with NHL or B-ALL (85, 86). Fludarabine conditioning chemotherapy proved effective in improving ORR (86). Clinical trials involving anti-CD19-CAR T-cells have shown better clinical responses in patients with ALL and chronic lymphocytic leukemia (CLL) when combined with cyclophosphamide conditioning (57, 87). Relapses were observed due to the low *in vivo* persistence of CAR T-cells and the emergence of CD19-negative cells as a mechanism of immune escape (57, 87). Interestingly, reports also showed the efficacy of anti-CD19 FMC63-28Z CAR T-cells alone in treating patients with ALL, CLL, DLBCL, and MCL, in the absence of prior chemotherapy (88). Graft-versus-host disease (GVHD) was observed in one patient (64, 88). Anti-CD19 CAR T-cells therapies have shown promising results when used as adjuvant treatments following autologous or allogeneic hematopoietic cell transplantation (HCT) in patients with ALL or B-cell NHL, with the former resulting in a higher ORR and 30-month PFS rate than allogeneic HCT (89). Phase I and II trials of axicabtagene ciloleucel, anti-CD19 CAR T-cells with CD28 costimulatory domain, have demonstrated anticancer response in refractory NHL when combined with cyclophosphamide and fludarabine chemotherapy, with an ORR of 82% and complete response rate of 54% in more than 100 treated patients (90). Similarly, anti-CD19 CAR T-cells with a 4-1BB costimulatory domain in combination with the aforementioned chemotherapy (90), resulted in an impressive ORR of 80% and a complete response rate of 60% in patients with lymphoma (91). Clinical trials using this combination therapy in patients with DLBCL are underway (92). While CD20-specific second-generation CAR T-cells containing a 4-1BB costimulatory domain were able to drive refractory DLBCL into partial remission when administered with prior conditioning chemotherapy (93), a phase II trial using the same CAR T-cells resulted in complete remission in six out of 11 patients with NHL (FL, MCL, DLBCL) patients (94).

Recent efforts in CAR T-cell development have targeted the identification of novel B-cell surface markers to improve selectivity of the therapy toward tumor cells, thereby sparing normal cells and reducing the side effects of CART-cell therapy. Three attractive targets, CD23 (present on CLL cells) (95), ROR1 (present on CLL and MCL) (96), and immunoglobulin kappa (κ) light chain (present on MCL, DLBCL, and some other NHLs) (97) are being evaluated for their anticancer activity in preclinical models since they are either not expressed or present at low levels in normal cells. CD22 is another potential target antigen expressed on B-ALL and other B-cell lymphomas (98). Preclinical results have demonstrated potent antitumor activity when at monoclonal antibody targeting a proximal epitope on CD22 is used for CAR T-cell production (98).

##### 3.2.2.2 T-Cell lymphoma

While TCL accounts for only a small proportion (~15%) of all NHL cases, they are associated with a worse prognosis compared to B-cell NHL (77, 99). Currently, therapeutic options for the treatment of TCL are limited to allogeneic HCT (100). Developing CAR T-cell therapies can be a breakthrough; however, it is imperative to do so by identifying antigen markers that are exclusively present on malignant T cells. One potential target antigen could be CD30 since some TCLs such as ALCL express it on their cell surfaces (68). Although high cytotoxicity was observed, natural killer cells have shown antitumor activity in preclinical T-cell ALL-derived cell lines (101). This study suggests that CAR T-cell therapies have the potential to treat complex, difficult-to-treat diseases. However, a better understanding of cytotoxicity management is required to improve the effectiveness of these therapies.

#### 3.2.3 Acute myeloid leukemia

In 2019, acute myeloid leukemia (AML) accounted for <2% of all cancers in the United States (56). The disease is associated with a poor prognosis owing to the limitation in finding a suitable target that is only present in AML cells and absent in normal hematopoietic stem cells (102). CD123, a hematopoietic cell marker, has shown efficacy in preclinical models (102, 103). A phase I clinical trial is currently ongoing to determine the safety and efficacy of second-generation autologous or allogeneic anti-CD123 CAR T-cells (with a CD28 costimulatory domain) in combination with cyclophosphamide and fludarabine chemotherapy (104). In addition to CD123, CAR-T cells specific for CD33, another myeloid antigen, have also shown promise *in vivo* for refractory AML (105). Higher expression of CD33 on normal cells makes them a less attractive target for treatment than CD123 (105). A phase I clinical trial, involving anti-Lewis Y (LeY) CAR T-cells with a CD28 costimulatory domain, demonstrated modest responses in two patients who had received prior fludarabine chemotherapy (106). CAR T-cells show durable persistence in patients, leading to mild toxicity (106). Other potential CAR T-cell therapy targets, including CD47, CD96, and CD44v6, are currently being investigated in preclinical models (100).

#### 3.2.4 Multiple myeloma

In 2019, 176,404 new multiple myeloma (MM) cases and 117,077 MM-related deaths are estimated worldwide (67). In the United States, in 2019, MM accounted for <2% of all cancers (56). MM cells express plasma cell surface antigens CD138 and CD38 (107). A phase I clinical trial involving CD138-specific CAR T-cells demonstrated efficacy with tolerable toxicities in five patients with refractory MM, with 4 patients reaching a stable disease state and one demonstrating a marked reduction of MM cells in the peripheral blood (108). Another phase I trial is ongoing to determine the dose-limiting toxicities associated with anti-CD138 CAR T-cell therapy in relapsed or refractory MM (NCT03672318).

B-cell maturation antigen (BCMA) is another surface marker present in B, plasma and MM cells (109). A clinical trial of anti-BCMA CAR T-cells with CD28 costimulatory domain conducted at NCI demonstrated partial responses in two patients and stable disease in 10 patients when treated with low doses of cells in combination with chemotherapy (110). High doses of CAR T-cells resulted in complete response in one patient and partial response in the other (110). Patients also experienced a higher degree of toxicity with increasing CAR T-cell doses (110). Anti-BCMA CAR T-cells alone have also shown efficacy in the absence of chemotherapy, leading to partial response in one patient and complete response in another, with toxicity levels similar to those observed in the NCI trial (111). MM cells demonstrate a classic immune escape strategy through the emergence of BCMA-negative cells (111). The infusion of low doses of anti-BCMA CAR T-cells with the 4-1BB costimulatory domain after chemotherapy resulted in partial response and mild toxicities in one patient, while high doses resulted in partial or complete responses in 11 out of 15 patients (112). A phase III trial is currently ongoing to determine the safety and efficacy of bb2121 in combination with standard MM treatment regimens and chemotherapy (**Table 2**). Another phase I trial with anti-BCMA CAR T-cells called LCAR-B38M has resulted in partial or complete responses with mild toxicities in 18 of the 19 treated patients (113). Anti-CD19 CAR T-cells administered to a patents with refractory MM following melphalan chemotherapy and autologous stem cell transplantation resulted in a complete response (114).

**Table 2:** Ongoing and currently recruiting phase III clinical trials involving CAR T-cell therapies for hematologic malignancies (22).

Intervention	Condition	Location	ClinicalTrials.gov Identifier
Anti-CD19 CAR T-cells with concurrent BTK inhibitor for BCL	BCL	Union Hospital, Wuhan, Hubei, China	NCT05020392
CAR-transduced autologous T cell intravenous infusion in subjects with R/R DLBCL with chemotherapy	R/R DLBCL	Multi-center study	NCT03391466
Anti-CD19 CAR T-cells with chemotherapy or blinatumomab in adults with B-ALL	B-ALL	Multi-center study	NCT04530565
BiRd regimen combined with BCMA CAR T-cell therapy in patients with MM	MM	The First Affiliated Hospital of Soochow University Suzhou, Jiangsu, China	NCT04287660
VRd regimen combined with autologous BCMA CAR T-cell therapy in patients with MM	MM	Multi-center study	NCT04923893
Autologous CAR T cell therapy targeting BCMA	MM	Multi-center study	NCT04181827
Efficacy and Safety Study of bb2121 Versus Standard Triplet Regimens in Subjects with R/R Multiple Myeloma (RRMM)	MM	Siteman Cancer Center, Saint Louis, MO, USA Hackensack University Medical Center, NJ, USA Sarah Cannon Research Institute Center for Blood, TN, USA	NCT03651128
Intravenous autologous CD19 CAR T-Cells for R/R B-ALL	R/R B-ALL	UKM Medical Centre Bandar Tun Razak, Kuala Lumpur, Malaysia	NCT03937544
Tisagenlecleucel in adult patients with aggressive B-cell NHL	B-cell NHL	University of Chicago Medical Center, Hematology & Oncology, IL, USA Sarah Cannon, Research Institute, TN, USA	NCT03570892

Preclinical evaluation of other potential antigen targets for CAR T-cell therapy such as CD38, CD44 isoform variant 6 (CD44v6), CD70, CD56, immunoglobulin κ light chain and signaling lymphocyte–activating molecule F7 (SLAMF7) is underway (115).

Currently, several phase III clinical trials are ongoing to determine the efficacy of CAR T-cells therapies targeting various antigens in combination with chemotherapy in patients with ALL, MM, AML and BCL (**Table 2**).

CAR T, chimeric antigen receptor-T; R/R, relapsed or refractory; B-ALL, B-cell acute lymphoblastic leukemia; B-LLy, B-cell lymphoblastic lymphoma; BCL, B-cell lymphoma; DLBCL, Diffuse Large B Cell lymphoma; MRD, minimal residual disease; CLL-1, C-type lectin-like molecule-1; AML, acute myeloid leukemia; MM, multiple myeloma; NHL, non-Hodgkin lymphoma; BCMA, B-cell maturation antigen.

## 4 Side effects of CAR T-Cell therapy

CAR T-cell therapies are known to cause severe side effects in various malignancies including CRS, GVHD, tumor lysis syndrome (TLS) and immune effector cell associated neurotoxicity syndrome (ICANS) (82, 116–119). CRS is activated by a massive increase in serum cytokine levels followed by T-cell activation (58, 65, 120) and is accompanied by nausea, vomiting, headaches, fever, myalgia, anorexia, coagulopathy, hypotension, renal dysfunction, and pulmonary edema (118). Severe CRS has been reported following by the administration of anti-CD19 CAR T-cell therapies in patients with NHL (86). A study conducted by Grupp et al. demonstrated the potential of tocilizumab, an anti-IL6 receptor antibody, in rapidly eliminating CRS (82).

Neurological toxicities may lead to B-cell aplasia, confusion, unresponsiveness, and seizures (118, 121), especially when anti-CD19 CAR T-cell therapies are administered in patients with lymphoma (86, 89). However, the mechanisms underlying these toxicities remain unknown (119). Notably, CRS and NS rates were higher in patients with hematologic malignancies than in those with solid tumors (60).

GVHD is often experienced by patients following the infusion of allogeneic lymphocytes from HTC donors, because of the response elicited by non-cancerous cells (122). Allogeneic anti-19 CAR T-cells cause chronic GVHD but no acute GVHD in patients with various B-cell lymphomas (118). The lack of GVHD may be attributed to the low persistence of CAR T-cells (76).

TLS is characterized by hyperkalemia, hyperuricemia, hypocalcemia, and hyperphosphatemia (118). Severe TLS has been observed in patients following infusion of anti-CD19 CAR T-cell therapies in various studies (59, 64).

Other less common side effects of CAR T-cell therapies include hypotension (87), pulmonary toxicity (123), hemorrhagic events (86, 93), and even death in rare cases (124). Strategies to eliminate CAR T-cells once the desirable response is achieved, are urgently required. Several studies have reported the use of biodegradable CAR T-cells, addition of an EGFR on the T-cell surface to be targeted by anti-EGFR antibodies, RNA electroporation (125, 126) or suicide gene incorporation (using target epitopes from CD34/CD20/caspase 9) (102, 103, 127–131). Although these approaches may work well, they should be used with caution since, the antitumor response achieved in patients may be affected in the absence of CAR T-cells (76).

## 5 Current challenges in CAR T-Cell therapy

The major challenges in the field of CAR T-cell therapy are to improve the *in vivo* persistence of CAR T-cells and identify ways to mitigate therapeutic toxicity. In addition, many unknowns in the field remain to be investigated, such as the mechanism of target-cell death, optimal dose needed for maximum efficacy, duration of *ex vivo* T-cells expansion, and efficacy of single vs multiple infusions of CAR T-cells.

CAR T-cells must persist and remain functional for a long time to prevent relapse. Long-term persistence of anti-CD19 CAR T-cells has been demonstrated in patients for many years after infusion (65, 82, 132). The limiting factors for *in vivo* CAR T-cell persistence may include *ex vivo* conditions in which T cell expansion occurs, stability of transgene expression, and immune responses developed against the transgene (133). Similarly, severe toxicities associated with CAR T-cell therapy may be due to the disease burden (84), high-dose chemotherapy regimen (87), high-dose CAR T-cell infusion (86), and as peak levels of serum cytokines and C-reactive protein (83, 86).

Determining the mechanism underlying target cell death, which may be caused by signaling domains associated with antigens or TCR complex chain, is crucials (134). The fate of the residual natural TCR remains unclear. T cells can also mediate target-cell death *via* granzyme release, cytokine release, and other immune effectors.

Responses to different doses of CAR T-cell therapy vary on a patient-by-patient basis. Some patients can greatly benefit from small doses, while others may not show any effect after infusion of a large dose. Therefore, it is challenging to determine the optimal T-cells dose for individual patients. Other important factors that may modulate this response are disease burden and toxicity levels (65, 82). A few studies recommend infusion of less than 10^8^ CAR T-cells following lymphodepletion in clinical trials to achieve a higher complete response rate (60, 135). Although infusion of multiple small doses of CAR T-cells has not shown any toxicity, it is still unknown whether single or multiple infusions lead to optimal efficacy remains unknown (59, 136).

The duration for which T cells need to be expanded in culture before infusion remains unclear. Since a less differentiated and more proliferative phenotype (such as T memory stem cells) is associated with better responses in preclinical models (137, 138), long-term *ex vivo* T cell expansion may not yield optimal results. Several crucial details regarding T-cell trafficking after infusion are currently unknown. Homing and trafficking of molecules on tumor vessels play a key role in modulating T-cell recruitment into the tumor microenvironment (139), thereby influencing the response in patients (140).

## 6 CAR T-Cell-derived nanovesicle therapy

Extracellular vesicles (EVs) are nano-sized membrane based-vesicles secreted by almost all cells and consistof exosomes (small EVs), microvesicles, apoptotic bodies and larger vesicles. EVs are capable of carrying various biological cargoes such as lipids, proteins and nucleic acids and resembles of their origin cells compositions (141–145). They are involved in local or distal intercellular communication by interacting with or delivering biologically active cargoes to recipient/target cells (146, 147). Immune cells such as dendritic cells, natural killer cells, macrophages, B- cells, and T-cellshave been shown to release EVs and are capable of modulating immunoregulation, tumor microenvironment and EV-based immunotherapy for cancers (148–152).

As EVs are mirror images of their parent cells in terms of their composition, CAR T-cell-derived EVs may substitute CAR T-cells and overcome some limitations. For example, CAR T-cells can proliferate in an uncontrolled manner thus inducing cytokine release syndrome (58, 123), which can lead to complications and even death (153),whereas EVs are non-proliferative biological nano-materials. Unlike cell therapies, EVs may not cause immune rejection (154). Immunotherapies can be hampered by tumor microenvironments; however, EVs are not influenced by the tumor microenvironments (155, 156).

**Figure 3:**
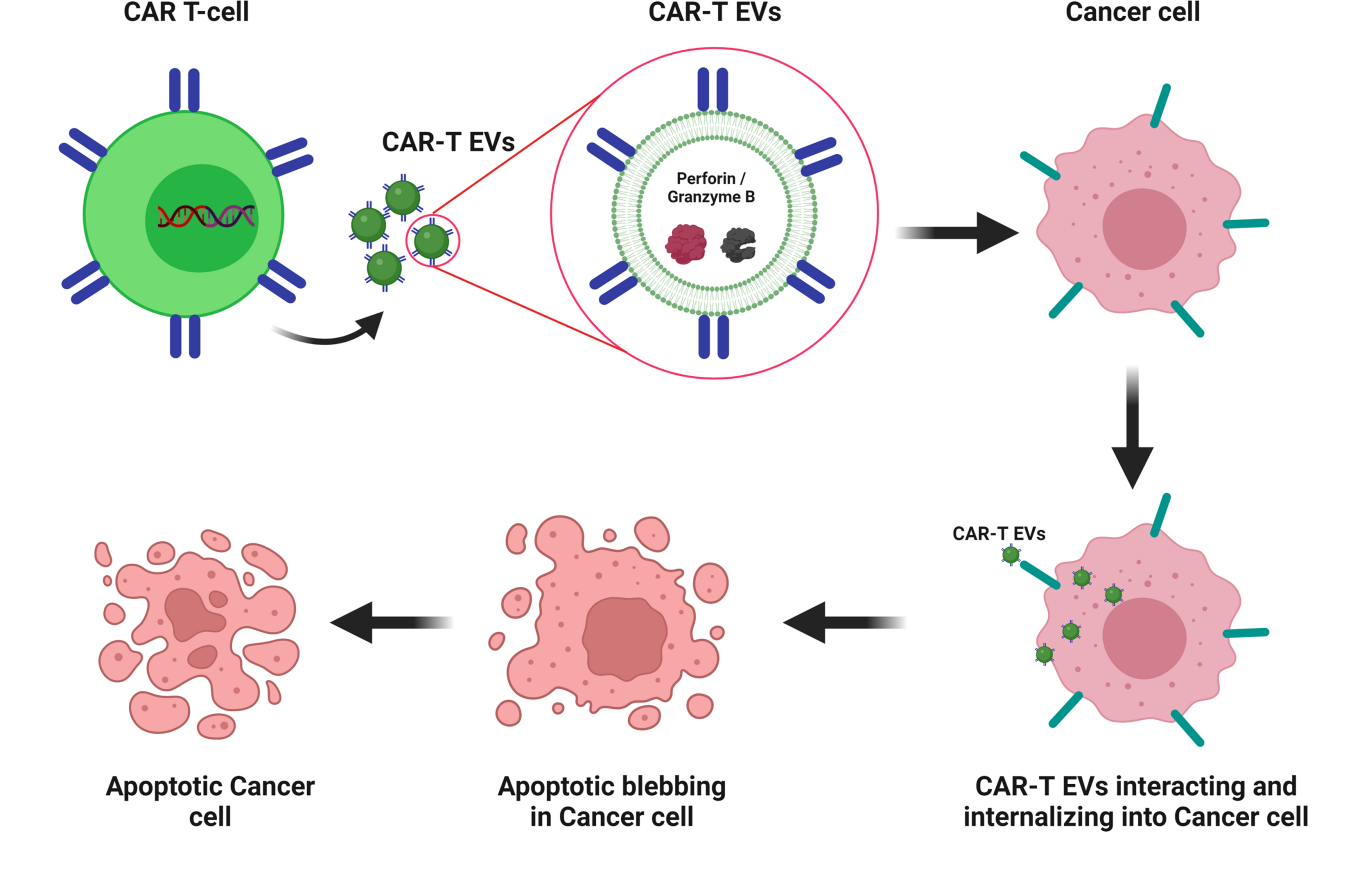
CAR-T EV-based therapy for cancer CAR-T EVs containing catalytic proteins (perforin and granzyme B). CAR-T EVs’ interacting and internalizing into cancer cells and leading to apoptotic blebbing and apoptosis. Created with BioRender.com.

Recent studies have reported the use of CAR T-cell derived EVs (exosomes or EVs) in cancer therapies (157–159). Exosomes derived from CAR T-cells (CAR-T exosomes) have shown high levels of cytotoxic molecules, such as perforin and granzyme B. CAR-T exosomes inhibit the growth of human breast tumors. Moreover, an *in vivo* preclinical model showed that the administration of CAR-T exosomes is safer than CAR-T cell therapy (157). Another study compared the penetration and cytotoxic activities of stimulated Anti-HER-2+ CAR T-cells and their CAR-T EVs. CAR-T EVs contain lower interferon gamma levels than CAR T-cells. Granzyme B levels were approximately 20-fold higher in CAR-T EVs than in EVs from unstimulated CAR T-cells. Anti-HER-2+ CAR-T EVs targeted HER-2 expressing cells. CAR T-cells showed more rapid cytotoxicity than their EVs (159). HEK293T cells were transduced with CD19 CAR plasmids, and their exosomes (Exo-CD19 CAR) were used to treat CD19 B-lineage leukemia. The results showed that Exo-CD19 CAR treatment induced cytotoxicity in CD19-positive leukemia B-cells but not in CD19-negative cells (158). These studies support the therapeutic use of EVs derived from CAR T-cells as a cell-derived nanovesicle-based therapeutic approach against tumors (**Figure 3**).

## 7 Strategies to overcome current clinical challenges associated with CAR T-Cell therapies

CAR T-cell persistence is major challenge faced by the CAR T-cell research community. Some of the strategies that can improve T cell persistence include administration of cytokines such as IL2, IL7, and IL15, and upregulation of proliferative or anti-apoptotic signals (87, 160, 161). In contrast, some studies have found that skipping IL-2 during CAR T-cell production resulted in higher ORR in patients with solid tumors and hematologic malignancies (60, 135).

Optimization of the CAR design is equally important for better persistence and overall treatment efficacy. Second-generation CARs have been shown to improve persistence compared to first-generation CARS;however, it remains unclear whether third-generation CARs are better at improving persistence than those in the second-generation CARs (81, 162). Among the different costimulatory molecules, CD137 and 4-1BB seem to work better than CD28 molecules in enhancing persistence and tumor trafficking, thereby improving the antitumor response in preclinical models (163, 164). Changes in the hinge and transmembrane regions of CAR regulate cell death and cytokine production (98, 165). A fully human CAR construct (HuCAR-19), designed to reduce immunogenicity and improve persistence (76), has shown an 86% ORR in patients with NHL in a first-of-its-kind clinical trial (166, 167). Clinical trials using two fully humanized CAR constructs are currently underway in patients with CD30+ NHL and HL as well as in those with CD19+ ALL and NHL. Preclinical studies have suggested an improved antitumor response when pharmaceutical agents and conditioning chemotherapy are administered in combination with CAR T-cell therapy (86, 168).

Tumor cells modulate the antigen expression on their cell surface to facilitate immune escape (57, 82, 87, 111). Therefore, CAR T-cells can no longer recognize and kill these cells. The efficacy of CAR T-cell therapy can be enhanced, and toxicity can be minimized by incorporating molecules specific for two or more target antigens, as demonstrated by some preclinical studies (169, 170). CAR T-cell therapies in conjunction with immune-checkpoint blockade are currently being investigated in patients with refractory or relapsed NHL (171).

Therefore, safer and cheaper gene transfer approaches are needed to reduce the overall cost of CAR T-cell therapy. While non-viral approaches, such as Sleeping Beauty, are inexpensive compared to lentiviral/retroviral vector-mediated gene transfer, there is a growing body of clinical evidence using the latter approach (172, 173).

Finally, CAR T-cell therapies have also been applied much later during the course of disease progression usually following chemotherapy, hematopoietic stem cell transplantation, or other treatments. The tremendous potential of applying CAR T-cell therapy at the beginning or earlier during the treatment course was unraveled and the strategy revealed higher success rates and reduced toxicity associated with anticancer treatments (174). Early administration of the therapy earlier may also give us access to a higher proportion of naïve, unexposed T-cell populations to facilitate the production of CAR T-cells.

## Author contributions

MJ, PG, and B-CA contributed to the conception, writing, and discussion of this manuscript. MJ, FK, CD, RR, and PG wrote the initial draft of the manuscript. B-CA Contributed to the critical conclusion of the manuscript. The final version of the manuscript has been approved by all authors.

## Funding

This work was supported by the National Research Foundation of Korea (NRF) grant funded by the Korean government (Ministry of Science and ICT) (NRF-2022R1A2C2005057).

## Conflict of interest

The authors declare that the research was conducted in the absence of any commercial or financial relationships that could be construed as a potential conflict of interest.

## Publisher’s note

All claims expressed in this article are solely those of the authors and do not necessarily represent those of their affiliated organizations, or those of the publisher, the editors and the reviewers. Any product that may be evaluated in this article, or claim that may be made by its manufacturer, is not guaranteed or endorsed by the publisher.
